# PPARs in Lung Biology and Disease

**DOI:** 10.1155/2007/28765

**Published:** 2007-11-25

**Authors:** Theodore J. Standiford, Jesse Roman

**Affiliations:** ^1^Division of Pulmonary and Critical Care Medicine, University of Michigan Medical Center, 4062 BSRB, 109 Zina Pitcher Place, Ann Arbor, MI 48109-2200, USA; ^2^Division of Pulmonary and Critical Care Medicine, Emory University School of Medicine, Whitehead Biomedical Research Building, 615 Michael Street, Suite 205-M, Atlanta, GA 30322, USA

In this special issue of PPAR Research, we have assembled a comprehensive and complementary group of review articles and original investigations that illustrate the pivotal role of the PPAR family of nuclear hormone receptors in the regulation of fundamental cellular events in the lung. The lung performs the vital function of gas exchange required for the delivery of oxygen to tissues. In performing this function, the lung is continuously exposed to a diverse array of microbial pathogens, allergens, and toxic particulate matter. These insults require immune and reparative responses that are appropriate, yet tightly regulated in order to maintain the delicate alveolar structures required for efficient gas exchange. PPARs and their obligatory heterodimer partners retinoid X receptors (RXR) are well known to regulate the expression of genes involved in lipid and glucose metabolism. More recently, these transcription factors have been shown to modulate developmental, inflammatory, and reparative responses, including those that occur in the lung.

Articles included in this special issue highlight the importance of PPARs and RXR in lung biology and in the pathogenesis of lung disease.Both PPAR-γ and RXR participate in lung morphogenesis, including the processes of postnatal alveolar elastogenesis and maturation.The majority of studies reported to date support a role for PPAR-α and/or PPAR-γ in inhibiting the release of inflammatory mediators from lung immune and stromal/parenchymal cells in vitro, and dampening inflammation and damage in animal models of acute lung injury (ALI), ischemia-reperfusion injury, and allergic airways inflammation. Emerging evidence indicates that PPAR family members can also suppress proliferative and differentiation responses of lung epithelial cells, smooth muscle cells, and fibroblasts, which is of particular relevance to tissue remodeling and fibroproliferation that occur in chronic airways disease, ALI, pulmonary vascular disease, and pulmonary fibrosis. In contrast to these notable inhibitory effects, PPAR-γ can also activate key macrophage antimicrobial and reparative responses, in part by enhancing the expression of cell surface receptors required for ingestion of microbes and cellular debris present within the airspace. Finally, data is summarized to illuminate the central role of PPAR-γ in regulating critical aspects of lung tumor initiation, progression, and metastasis. Specific effects include promotion of tumor cell differentiation, induction of cell cycle arrest and apoptosis, suppression of angiogenesis, and modulation of immune/stromal cells within the tumor microenvironment.

The field of PPAR research continues to be hindered by the nonselective effects of synthetic and naturally occurring agonists, the limited availability of potent and specific inhibitors, and the absence of ideal genetic models of PPAR deficiency. The introduction of new molecular tools and the generation of conditionally targeted and site specific (including lung epithelial cell-specific) PPAR-γ deficient mice will allow better distinction between PPAR-dependent and PPAR-independent effects. Moreover, the search for relevant endogenous PPAR ligands continues. The recently described nitroalkene species are appealing candidates, although the presence of these molecules in lung tissues has not yet been characterized.

We thank the editors for the opportunity to share with the readership this important area of investigation. It is our hope that this special issue will serve as a catalyst for new initiatives in this exciting and rapidly evolving field. Observations made at the bench have already transitioned to the bedside, as trials assessing effects of PPAR-γ agonists in the treatment of diverse lung diseases, including asthma and lung cancer, are ongoing.

*Theodore J. Standiford* *Theodore J. Standiford* *Jesse Roman* *Jesse Roman*

